# New Appliances and Things Medical

**Published:** 1895-10-05

**Authors:** 


### NEW APPLIANCES AND THINGS MEDICAL.

[We shall be glad to receive, at our Office, 428, Strand, London, W.C., from the manufacturers, specimens of all new preparations and appliances which may be brought out from time to time.]

### SURGICAL DRESSINGS, &c.

(Seabury and Johnson, 32 and 33, Snow Hill, E.C.)

We have just examined a number of samples of the surgical dressings issued by this firm. Manufacturers in general may learn much by a study of their methods. All their preparations are most attractively "got up," and almost without exception are unsurpassed as regards quality and finish. Messrs. Seabury and Johnson make a special feature of supplying no medicated preparations that have not been carefully standardised and assayed by qualified chemists. The percentage of the antiseptic is based on the weight of the finished dry product, and is plainly named upon the label. Their standard gauzes, of which there is a large variety, including Lister's eucalyptol, hydronaphthol, and thymol, are supplied in five-yard containers, and folded by a patent process so that as much or as little as required can be removed without exposing the rest to sources of contamination. The containers above mentioned are of most useful design, being air-tight and of simple structure. They received a special award at the Chicago Exhibition, 1893. The B. W. B. elastic woven bandage is likely to replace the older forms, which have usually been found unbearably hot in warm weather; for varicose veins and in other cases in which equable pressure is necessary they will be found particularly useful. Space does not allow us to mention all the special features in these preparations, but practitioners will do well to peruse the catalogue and price list, as they will discover many useful dermatological and antiseptic plasters, as well as other sick-room adjuncts most valuable for private practice. In fact, this firm appears to be a veritable caterer of invalid luxuries.

### BEEF PEPTONE.

(Liebig's Extract of Meat Company.)

Liebig instructed the world in the methods of invalid feeding, and in the physiological economy of concentrated meat extracts. The vicissitudes of the original preparation which bears his name are now matters of history, but public interest is once again attracted to the question by our enlightened knowledge of the nutritive value of the contained albumoses and peptones, and by the results of recent and more complete analyses. There was a critical period in the history of all meat extracts, when these preparations threatened to fall into disfavour, owing to the absence or deficient percentage of albumen, which alone of the proximate principles of meat essences was regarded by physiologists as of nutritive value. From insufficient laboratory knowledge the soluble albumoses and peptones were classed with the extractives and salts, and regarded as stimulants rather than as foods. Thanks to improved methods, we now know how to recognise the presence of these bodies, and appreciate them as of high nutritional value. The larger their percentage in any extract the more valuable the preparation. Liebig's extract shows by analysis a very high percentage of assimilable peptones and albumoses, thus scientifically confirming what empyrical experience has long taught, namely, that its clinical use in conditions of exhaustion or malnutrition is followed by marked improvement. Liebig's Company are now the first to take advantage of scientific criticism and alien success; in fact, they are going one better than their rivals in the trade, and are producing an extract of meat which at once contains an overwhelming percentage of peptones and albumoses, and at the same time is agreeable and bland to the palate. This new preparation is called peptone of beef, or peptonum carnis; it is a real food, a concentrated food, and one that can be immediately assimilated by the gastric mucous membrane, even in conditions of the weakest physiological activity. Further, it is a gastric stimulant, both direct and indirect, due to the presence respectively of extractive and refreshing aromatic principles.

### MALTOPEPTONE MALT EXTRACT JELLY AND COD-LIVER OIL.

(The Maltopeptone Company, Needham Market, Suffolk.)

The Maltopeptone Company have submitted to us samples of their Malt Extract Jelly, which possess several distinct features over other forms of malt extract which render it worthy of the notice of the profession. The term maltopeptone is, however, in our opinion badly chosen for designating this company's speciality, inasmuch as there is no true peptone added or incorporated with the malt extract, as was proved by the fact that a solution of the jelly in water when dialysed through a membrane failed to give the biuret reaction. The company, however, do not claim the presence of peptone in their preparations, but use the term maltopeptone to denote the extract of malt which they obtain by their special patented process, in which not only is the diastase extracted, but also the soluble nitrogenous matter and natural salts contained in the malt rootlets. This addition has a two-fold object; (*a*) it greatly adds to the nitrogenous constituents present, and thus increases its value as a nitrogenous food stuff. From a Kjeldahl determination of the nitrogen in a sample of the jelly our analyst found 1·45 per cent. of nitrogen, and in another sample containing cod-liver oil the total nitrogen amounted to 1·4 per cent; (*b*) the addition improves the flavour of the malt extract, which has none of the nauseating sickly taste which is noticed in some of the malt extracts of other makers. The diastasic value of the jelly examined by us in accordance with Lintner's process, in which a 5 per cent. solution of the jelly in water was tested, gave only 40 points on Lintner's scale, and although many commercial malt extracts do not give a higher figure than this, it shows that the company's method does not yield a product which can be considered exceptionally rich in diastasic activity.

The jelly and oil preparation was similarly tested and gave 30 points, showing that the addition of the oil had diminished the relative diastasic value of the preparation. The company's malt extract and cod-liver oil is made by the addition of 25 per cent. (whether by weight or volume is not stated) of cod-liver oil to the extract, but no mention of the amount of oil present in the jelly is mentioned. From several experiments by different methods of extraction the amount of oil found proved not to be greater than 11·5 per cent. by weight, and this result may be attributed either to the jelly initially containing a smaller quantity of oil than that which is stated to be present in the extract, or to the fact that the oil is so completely emulsified that the ordinary methods of extraction fail to recover the whole of the oil present. Both preparations seem to be very carefully manufactured, and may be adequately described as elegant, and we are of the opinion that the jelly form for administering malt and oil is to be recommended, and we hope that the profession will give this company's products a good share of their patronage.

